# Comparison of Different Modeling Approaches for Prescription Opioid Use and Its Association With Adverse Events

**DOI:** 10.1093/aje/kwad115

**Published:** 2023-05-15

**Authors:** Siyana Kurteva, Michal Abrahamowicz, Marie-Eve Beauchamp, Robyn Tamblyn

**Keywords:** adverse events, cohort study, Cox proportional hazards models, flexible-modeling, opioid prescribing

## Abstract

Previous research linking opioid prescribing to adverse drug events has failed to properly account for the time-varying nature of opioid exposure. This study aimed to explore how the risk of opioid-related emergency department visits, readmissions, or deaths (composite outcome) varies with opioid dose and duration, comparing different novel modeling techniques. A prospective cohort of 1,511 hospitalized patients discharged from 2 McGill-affiliated hospitals in Montreal, 2014–2016, was followed from the first postdischarge opioid dispensation until 1 year after discharge. Marginal structural Cox proportional hazards models and their flexible extensions were used to explore the association between time-varying opioid use and the composite outcome. Weighted cumulative exposure models assessed cumulative effects of past use and explored how its impact depends on the recency of exposure. The patient mean age was 69.6 (standard deviation = 14.9) years; 57.7% were male. In marginal structural model analyses, current opioid use was associated with a 71% increase in the hazard of opioid-related adverse events (adjusted hazard ratio = 1.71, 95% confidence interval: 1.21, 2.43). The weighted cumulative exposure results suggested that the risk cumulates over the previous 50 days of opioid consumption. Flexible modeling techniques helped assess how the risk of opioid-related adverse events may be associated with time-varying opioid exposures while accounting for nonlinear relationships and the recency of past use.

## Abbreviations

aHR: adjusted hazard ratio
AIC: Akaike information criterion
CI: confidence interval
ED: emergency department
MME: morphine milligram equivalent
MSM: marginal structural model
RAMQ: Régie de l'assurance maladie du Québec
TD: time-dependent
WCE: weighted cumulative exposure

Opioids are the most commonly prescribed medications for treating pain (1). Trends of increasing use of prescribed opioids have been accompanied by increases in opioid-related morbidity and mortality (2, 3). Recent research suggests that even short-term use may lead to increased risk of adverse effects (4).

Understanding how risks vary depending on the duration and/or consumption patterns is instrumental in developing evidence-based prescribing guidelines and public health strategies for addressing the growing opioid epidemic (5). Longer duration and higher doses of prescription opioids were associated with increased risk of severe adverse events such as opioid overdose, dependence, and abuse (6–12). Previous studies inadequately modeled the dynamic changes in patients’ comorbidities and concurrent medication use, which could possibly induce confounding by indication bias (13). Currently, no consensus exists regarding what duration of use is “safe” versus potentially harmful (14), and most published analyses relied on arbitrarily selected thresholds, such as 90 days (15–20). Yet the risks of adverse events may increase gradually with increasing cumulative dose and/or duration of past opioid use (21). Moreover, conventional regression does not consider: 1) possibly nonlinear associations with the duration of use, and 2) the fact that the impact of past exposure may depend not only on the duration of past exposures but also on how recently they occurred.

To avoid such limitations, the overarching goal of the present study was to compare various statistical modeling approaches, including conventional and flexible techniques, to gain further insights regarding how risks of adverse events may vary depending on the current and past opioid use. First, we assessed the association between time-varying opioid exposures and the risk of opioid-related emergency department (ED) visits, hospital readmissions, or all-cause mortality in the 1 year after discharge, while accounting for duration, doses, and recency of past use. Second, we assessed whether the methodological approach used to model time-varying opioid exposures had an impact on the results and conclusions.

## METHODS

### Design and study population

A prospective cohort of medical and surgical patients was assembled from a cluster-randomized trial of medication reconciliation (RightRx) conducted at the McGill University Health Centre (MUHC) between October 2014 and November 2016 (22). To be eligible for the original trial, patients had to be 18 years or older at admission, with at least 1-year prior continuous provincial health-care coverage. To be included in this study, patients needed to fill at least 1 opioid prescription in the 90 days after discharge. We excluded patients with history of using methadone or buprenorphine, often given to treat opioid addiction (Web Figure 1, available at https://doi.org/10.1093/aje/kwad115) (18).

### Data sources

Demographic and clinical information, health-care service use, and dispensations were retrieved from the admission notes and the provincial health-care (Régie de l'assurance maladie du Québec, or RAMQ) administrative medical services and prescription claims databases in the year prior to and the year after the hospitalization. Admission and discharge dates and units, patient health problems, and major procedures were retrieved from the hospitalization database. Diagnoses and medications taken—1) at admission, 2) in hospital, 3) prescribed at discharge, and 4) dispensed in the community after discharge—were abstracted from the RAMQ prescription database and MUHC Data Warehouse, one of the few databases in the world that links information on all 4 sources of medication use. Hospital discharge experience data were obtained via telephone interviews 30-day after discharge. Ethics approval was provided by the MUHC Research Ethics Board. Privacy Commissioner approval was obtained to link clinical and administrative data from the Commission d’accès à l’information du Quebec.

### Outcomes

We used a composite endpoint of the earliest of the first opioid-related ED visit and/or readmission, or all-cause death, in the 1 year after discharge. Outcomes were ascertained using RAMQ provincial medical services claims and hospitalization databases, to ensure that all ED visits and readmissions were included. An adverse event was considered opioid-related if there was a diagnosis of opioid abuse, opioid dependence, and/or opioid poisoning at the time of the ED visit or hospitalization, and/or there was a recorded diagnosis of 1 or more common opioid side effects: constipation, nausea, vomiting, dizziness, or fractures (23–27). The choice of the broader outcome was necessary because only 3 patients (0.02%) experienced opioid-specific events such as opioid abuse, opioid dependence, and/or opioid poisoning.

### Opioid use

Time-varying opioid use 1 year after discharge was measured using RAMQ prescription claims, which document, for each prescription filled, the drug identification number (DIN), strength, dispensing date and quantity, and duration. Web Appendix 1 and Web Table 1 provide details of how exposure status and dose were reconstructed. DINs mapped to the Anatomical Therapeutic Chemical (ATC) codes N02A, R05DA were used to identify opioids. Daily dose was converted to morphine milligram equivalent (MME) doses using the Centers for Disease Control and Prevention Opioid Morphine Equivalent Conversion Factor and the concurrently dispensed doses were added together (28). Unique to this study, administratively derived measures of opioid exposure were supplemented with information on medication-taking behavior, extracted from patients’ interviews, to identify who filled a prescription and who: 1) continued taking it, 2) started taking it but stopped, or 3) never used it.

### Opioid exposure metrics

As the potential biological mechanisms that relate past and/or current opioid use to an increased risk are not known, it is important to consider alternative metrics to account for the complex time-varying nature of exposure and compare their results (29). Otherwise, exposure mismodeling may lead to etiologically incorrect conclusions, including failure to detect an association due to exposure or inability to discriminate between short-term acute versus lagged or cumulative exposure effects (29). Thus, we used 4 alternative time-varying metrics of opioid use, updated every day of follow-up: 1) cumulative or 2) continuous duration of past opioid use, 3) current daily opioid dose, and 4) current opioid use (see Web Table 2).

### Potential confounders

Potential risk factors for long-term opioid use and opioid-related harms were identified from the literature (20, 30–37). The baseline time-invariant covariates included demographic characteristics and health-care utilization in the year prior to hospitalization, reasons for hospitalization, and in-hospital opioid use. Time-varying indicators of physical and mental health comorbidities that are likely associated with increased pain, and thus may lead to increased opioid use, were also constructed (18, 38). (Web Table 1 lists all covariates.) To distinguish between those who may use opioids for acute vs. other types of pain, surgical or internal medicine patients were identified based on the discharge unit.

### Statistical analyses

Descriptive statistics were used to summarize baseline characteristics. For main analyses, we used multivariable marginal structural Cox proportional hazards models (MSM) (39, 40) and their flexible extensions. This allowed us to estimate the associations between time-varying opioid use and the time to the composite outcome, while controlling for time-varying confounders possibly affected by prior opioid exposure. To avoid immortal time bias (41), time zero corresponded to the start of the first postdischarge opioid dispensation, and patients who had no events until 1 year after discharge were censored at that time. Time-varying and time-fixed covariates were used to estimate time-varying stabilized inverse probability treatment weights (42, 43) for opioid exposures (see Web Table 1 for covariates included), updated at 10-day intervals and truncated at the 95th percentile (43).

For each exposure metric, we first fitted the conventional marginal structural Cox proportional hazards model, which imposed 2 restrictive assumptions: the linearity assumption, which implied that the logarithm of the hazard was a linear function of a continuous exposure metric, and the proportional hazards assumption, which forced hazard ratios to be constant across follow-up (44). Yet either or both assumptions may often be violated. To avoid these restrictive assumptions, we employed a flexible extension of marginal structural Cox models to reassess the roles of: 1) cumulative and 2) continuous duration of use, 3) current daily MME opioid dose, log-transformed because of a very skewed distribution, and 4) current opioid use. First, for each time-varying opioid exposure metric we tested: 1) the proportional hazards assumption and 2) for continuous exposure metrics, the linearity assumption (45). If at least 1 of these assumptions was rejected, at 2-tailed α = 0.05 for the respective likelihood ratio test (45), we relied on a flexible spline-based model to estimate, respectively, the: 1) time-dependent (TD) and/or 2) nonlinear effects of the corresponding exposure metric (46). The estimated TD effect describes how the strength of the association between a given exposure metric and the hazard changes during follow-up, whereas the nonlinear estimate indicates how the logarithm of the hazard varies with increasing values of a metric (46), and may possibly suggest an approximate threshold for the association of interest (47). In sensitivity analyses, to provide a comprehensive assessment of different aspects of exposure history, we estimated flexible models including simultaneously nonlinear effects for both current daily dose and either continuous or cumulative opioid use duration.

In addition, we hypothesized that the impact of past exposure may depend not only on the duration of past use or total past cumulative dose, but also on how recently past exposures occurred (21). Thus, we also used the flexible recency-weighted cumulative exposure (WCE) model, in which the cumulative effect of a time-varying exposure, at a given day during follow-up, is modeled as a weighted sum of: 1) past doses or 2) binary indicators of use at different days in the past (48). In contrast to conventional (unweighted) metrics of: 1) cumulative sum of all past doses or 2) total duration of past use, the time-varying WCE metrics assign differential weights to past exposures/doses. These weights depend on the time elapsed since exposure, and reflect the relative impact of doses taken(e.g., 1 week ago vs. 3 weeks ago) on the current hazard. The weight function is estimated using cubic regression splines (49).

In preliminary WCE analyses over the entire 1-year follow-up window, the estimated weights decayed to zero for exposures about 3 months prior and became increasingly unstable, as indicated by wide 95% confidence bands, for earlier exposures (Web Figure 2). Given these results and earlier simulations showing that using a slightly longer time window reduces the risk of biased WCE estimates (49), in the final WCE analyses we considered only opioid use within the most recent 120 days, with the weight function constrained to smoothly decay to zero at 120 days. We then fitted alternative WCE models of increasing flexibility/complexity, with 1–3 interior knots, and chose the best-fitting model based on the minimum Akaike information criterion (AIC) (49). Based on the final best-fitting WCE model, we estimated hazard ratios for prespecified, clinically relevant patterns of past opioid use, relative to no opioid use, in the previous 120 days (50).

Flexible and WCE MSMs were adjusted for the same time-fixed and time-varying covariates and using the same inverse probability treatment weights as in conventional MSM Cox analyses (51). The 95% pointwise confidence bands for nonlinear and TD estimates and WCE-based weight functions were obtained using bootstrap resampling (44, 49). AIC was used to compare the goodness of fit of: 1) flexible versus conventional models for the same exposure metric, and 2) models with alternative exposure metrics. An AIC difference of 4 or more points indicates that the model with lower AIC is more consistent with the underlying association between time-varying exposure and the hazard (29).

Marginal structural Cox models were implemented with R (version 3.4.2; R Development Core Team, Vienna, Austria), and flexible spline-based models were implemented with the CoxFlex function (52) for nonlinear/TD effects and the WCE package (53).

## RESULTS

The mean age was 69.6 (standard deviation, 14.6) years, with 57.7% of male sex (Table 1). Sixteen percent (*n* = 241) of the cohort had a potentially opioid-related ED visit or hospitalization or died in the 1 year after hospital discharge, with a mean time from discharge to the event of 129.7 days, and an incidence of 180.4 per 1,000 person-years. Fractures (55.4%), nausea and vomiting (17.7%), and dizziness (14.4%) accounted for most of the potentially opioid-related ED visits/readmissions.

Patients’ interviews revealed excellent adherence to opioid dispensation: 85% (*n* = 1,360) of patients reported taking their dispensed opioids as prescribed, only 11% (*n* = 169) discontinued their initial dispensation, and 4% (*n* = 70) never started taking dispensed opioids in the first month after discharge.

**Table 1:** Selected Characteristics of Patients Who Filled at Least 1 Opioid Prescription in the 3 Months After Discharge From McGill-Affiliated Hospitals in Montreal Between 2014 and 2016

			**Hospital Discharge Service**			
	**Overall (*n* = 3,486)**		**Internal Medicine (*n* = 392)**		**Surgery (*n* = 1,119)**	
**Variable**	**No.**	**%**	**No.**	**%**	**No.**	**%**
Age, years^a^	69.6 (14.9)		67.7 (16.8)		66.9 (11.9)	
Male sex	2,010	57.7	200	51.0	683	61.0
Length of hospital stay ≥6 days	2,930	84.0	351	89.5	875	78.2
No. of emergency department visits^b^	3.0 (10.0)		9.0 (18.5)		2.0 (6.0)	
No. of hospitalizations^b^	0.0 (1.0)		0.0 (1.0)		0.0 (1.0)	
No. of physicians prescribing opioids^b^	4.0 (6.0)		5.0 (6.0)		3.0 (3.0)	
No. of pharmacies dispensing opioids^b^	1.0 (2.0)		1.0 (1.0)		1.0 (1.0)	
Active opioid prescription at admission	504	14.5	186	47.4	105	9.4
History of opioid use	1,206	34.6	283	72.2	344	30.7
≥3 opioid dispensations	104	2.9	61	15.6	18	1.6
History of long-acting opioids	146	4.2	89	22.7	35	3.2
History of methadone/buprenorphine	13	0.4	10	2.6	1	0.1
Opioids	1,530	43.9	202	52.5	987	88.2
Nonopioid analgesics	2,209	63.4	227	57.9	990	88.5
Mental illness	511	14.7	74	18.9	132	11.8
Dementia	213	6.1	25	6.4	13	1.2
Substance and alcohol abuse	115	3.3	27	6.9	19	1.7
Pain syndromes	1,352	38.8	221	56.4	408	36.5
Cancer	1,253	35.9	168	42.9	538	48.1

^a^ Values are expressed as mean (standard deviation).

^b^ Values are expressed as median (interquartile range).

In conventional marginal structural Cox models, current opioid use was associated with a 71% increased hazard of the composite outcome of adverse events or all-cause mortality (adjusted hazard ratio (aHR) = 1.71, 95% confidence interval (CI): 1.21, 2.43) (Table 2). Compared with short duration of use (1–30 days), there were 2-fold hazard increases associated with past cumulative duration of opioid use of 60–90 days (aHR = 2.39, 95% CI: 1.34, 4.29) and more than 90 days (aHR = 2.61, 95% CI: 1.59, 4.27). Uninterrupted continuous use of 30–60 consecutive past days was also associated with a 2-fold increased risk (aHR = 2.57, 95% CI: 1.45, 4.55), compared with patients not currently using opioids). Compared with <90 MMEs, there was a 3-fold higher hazard for current daily opioid doses exceeding 90 MME (aHR = 3.51, 95% CI: 1.58, 7.82), indicating a dose-response relationship.

**Table 2:** Results From Conventional Marginal Structural Cox Models for the Association Between Different Opioid Exposure Metrics and Risk of Emergency Department Visits, Readmission, or Deaths for Patients Discharged Alive From McGill-Affiliated Hospitals in Montreal Between 2014 and 2016

**Opioid Exposure Metric**	**Average Starting Dose in MME, Mean (SD)**	**Average DailyDose in MME, Mean (SD)**	**CrudeHR**	**HR Stabilized Weights** ^**a**^	**95% CI**	**AIC**
Current opioid use						2 611.3
No	33.5 (19.3)		1.00	1.00	Referent	
Yes	41.2 (43.3)	57.1 (75.2)	3.95	1.71	1.21, 2.43	
Cumulative duration of opioid use, no. of days						2 606.4
1–30	33.7 (19.6)	41.3 (43.6)	1.00	1.00	Referent	
31–60	32.9 (24.7)	51.4 (71.5)	2.46	1.55	0.99, 2.41	
61–90	38.4 (35.5)	62.6 (85.0)	4.56	2.39	1.34, 4.29	
≥91	44.9 (48.2)	72.5 (91.2)	5.95	2.61	1.59, 4.27	
Continuous duration of opioid use, no. of days						2 611.4
0	33.5 (19.3)	0	1.00	1.00	Referent	
1–30	36.1 (29.6)	41.5 (44.9)	3.86	1.08	0.52, 2.23	
31–60	41.3 (44.4)	57.9 (79.6)	6.07	2.57	1.45, 4.55	
≥61	51.0 (59.9)	86.3 (104.6)	3.04	1.67	1.08, 2.58	
MME current daily dose (log-transformed)						2 588.2
<90	33.3 (19.8)		1.00	1.00	Referent	
≥90	93.5 (82.2)		5.72	3.51	1.58, 7.82	

Abbreviations: AIC, Akaike information criterion; CI, confidence interval; HR, hazard ratio; MME, morphine milligram equivalent; SD, standard deviation.

^a^ The 95th percentile for the stabilized weight was 2.88 (mean = 0.81 (SD, 0.71)). Covariates considered in the construction/calculation of the weights: 1) demographic characteristics (indicator for a patient randomized to the RightRx intervention group, age at admission, sex, copay status), 2) medical, prescription and health care use 1 year before admission (unique number of dispensing pharmacies and prescribers, hospitalizations and emergency department visits, the receipt of radiotherapy and/or chemotherapy services, type of cancer, history of mental health diagnoses, history of substance and/or alcohol abuse/dependence, targeted comorbidities that may increase someone’s risk of opioid-related adverse events, history of chronic pain, previous opioid use, more than 3 opioid dispensations, previous use of psychotropic medications), 3) in-hospital characteristics (presence of an opioid-related reason for index admission, length of hospital stay, opioid administration during the index hospitalization, nonopioid pain medication administration, use of antidepressant and benzodiazepines, hospital unit discharged from (medical vs. surgical), type of surgery (cardiac vs. thoracic)), 4) at discharge (receipt of an opioid prescription, prescribing reason (having had surgery, having anxiety or pain problems)), and 5) time-varying postdischarge characteristics (use of benzodiazepines, use of antidepressants, use of methadone/buprenorphine, cumulative number of physicians, cumulative number of dispensing pharmacies, recent discontinuation of opioid use, recent increases in opioid dose, recent add-on opioid therapy, updated targeted baseline medical comorbidities).

Among the 4 exposure metrics, the log-transformed current MME dose fit the data best for conventional marginal structural Cox models (lowest AIC, last column of Table 3). For all continuous exposure metrics (cumulative or continuous duration of use, and current daily dose), flexible modeling of the nonlinear effect improved the models’ fit to data, with very substantial AIC reductions of 15 or more points with respect to conventional marginal structural Cox models (Table 3). On the other hand, there was no evidence of TD effects of any of the exposure metrics (Table 3). Indeed, all *P* values for TD effects were above 0.079 when the significant nonlinear effects were accounted for, indicating that, consistent with the proportional hazards assumption, the strengths of the corresponding associations did not vary during the 1-year follow-up.

**Table 3:** Comparison of Goodness of Fit of Conventional and Flexible Marginal Structural Models With Alternative Time-Varying Opioid Exposure Metrics for Patients Discharged Alive From McGill-Affiliated Hospitals in Montreal Between 2014 and 2016

**Opioid Exposure Metric and Statistical Model**	**AIC**	***P* Value (Nonlinear)**	***P* Value (TD)**
Current use			
Conventional Cox MSM	2 611.3		
Flexible TD MSM	2 610.8		0.087
Flexible WCE MSM	2 591.4		
Cumulative duration of use			
Conventional Cox MSM	2 606.3		
Flexible nonlinear MSM	2 584.0	0.000	
Flexible TD MSM	2 609.6		0.007
Flexible nonlinear + TD MSM	2 590.9	0.000	0.897
Continuous duration of use			
Conventional Cox MSM	2 611.4		
Flexible nonlinear MSM	2 596.0	0.000	
Flexible TD MSM	2 620.5		0.136
Flexible nonlinear + TD MSM	2 595.6	0.000	0.079
MME current daily opioid dose (log-transformed)			
Conventional Cox MSM	2 588.2		
Flexible nonlinear MSM	2 570.5	0.001	
Flexible TD MSM	2 583.8	0.394	
Flexible nonlinear + TD MSM	2 577.7	0.007	0.928
Flexible WCE MSM	2 576.5		

Abbreviations: AIC, Akaike information criterion; MSM, marginal structural model; TD, Time-dependent; WCE, weighted cumulative exposure.

The nonlinear estimate in Figure 2 shows also a strong nonlinearity, with very steep risk increases within the first 2 weeks of recent continuous uninterrupted opioid use (Figure 2). However, the nonlinear model for cumulative duration shows a much better fit (12 points improvement in AIC, Table 3), indicating that it is a more relevant exposure metric. Finally, the nonlinear estimate in Figure 3 indicates that the risk increases continuously with increasing current log-transformed daily opioid MME dose, with steeper increases for higher doses. This model showed the best fit among all possible models and opioid exposures tested (Table 3).

**Figure 1:**
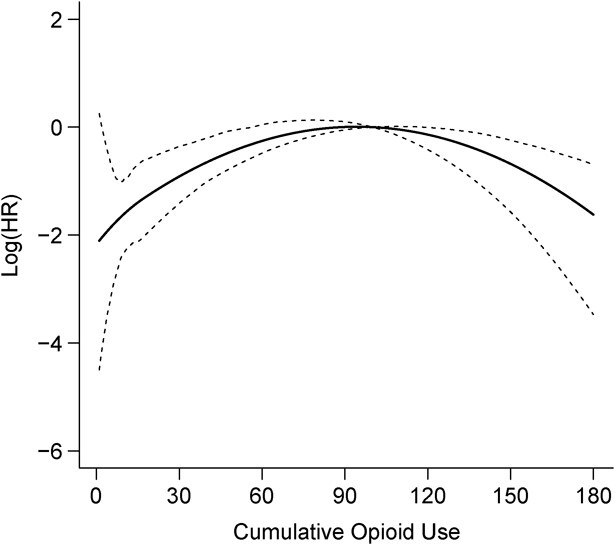
Nonlinear effect of cumulative duration of opioid use and the risk of opioid-related emergency department visits, readmissions, or deaths. Data on opioid use and health-care utilization was collected from patients discharged alive from the McGill University Health Centre (MUHC), Canada, between October 2014 and November 2016. The solid line represents the effect estimate of the nonlinear effect of cumulative use on the risk of opioid-related emergency department visits, readmissions, or deaths, and the dashed lines represents the corresponding confidence intervals associated with it. HR, hazard ratio.

The nonlinear estimate in Figure 1 shows that the risk of opioid-related adverse events increases gradually as total time-varying cumulative duration of past opioid use increases up to about 50–60 days. With further increases above 2 months of cumulative use, risk increases are only moderate and there is no evidence of further impact of durations longer than 100 days, when the curve reaches a plateau and the estimates become very imprecise with wide 95% CIs (Figure 1), because such long exposures were accumulated by only a few subjects. This flexible nonlinear MSM fits the data much better than the conventional linear Cox MSM (Table 3) and reveals much higher impact of increasing cumulative duration of past use beyond a few weeks (Figure 1).

**Figure 2:**
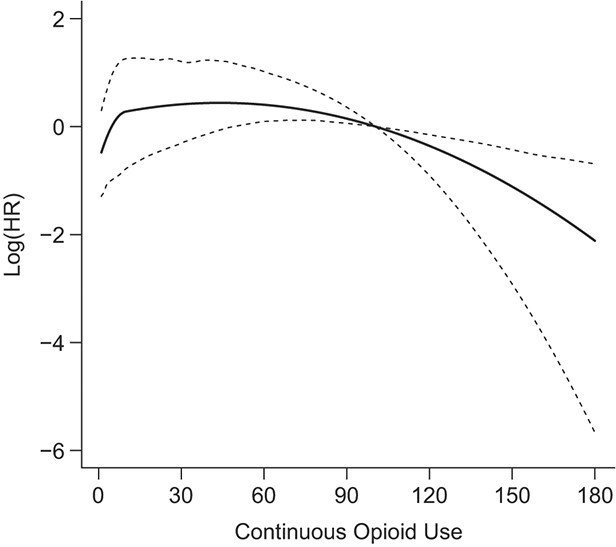
Nonlinear effect of continuous opioid use and the risk of opioid-related emergency department visits, readmissions, or deaths. Data on opioid use and health-care utilization was collected from patients discharged alive from the McGill University Health Centre (MUHC), Canada, between October 2014 and November 2016. The solid line represents the effect estimate of the nonlinear effect continuous opioid use and the risk of opioid-related emergency department visits, readmissions, or deaths, and the dashed lines represents the corresponding confidence intervals associated with it. HR, hazard ratio.

In sensitivity analyses, where the duration was additionally adjusted for the nonlinear effect of current daily dose, there were even greater AIC reductions of 65 points for continuous and 35 points for cumulative use (Web Table 3). The nonlinear estimates for cumulative duration of use are similar whether adjusted or not for daily dose (Web Figure 3). However, for continuous duration of use, the nonlinear estimate changes considerably when adjusted for daily dose and shows decreasing hazard for any increase in duration, even in the low range of first 2 weeks (Web Figure 4), in contrast to the estimate not adjusted for dose (Figure 2). This pattern of results suggests that, among patients on the same current dose, the hazard decreases with longer continuous opioids use, possibly due to improved tolerance and/or healthy survivor effect (54–58). As a corollary, this suggests that higher hazard for longer continuous durations (when not adjusted for current daily dose) may partly reflect higher daily doses of long-term users (Pearson *r* = 0.65 for correlation of daily dose with continuous duration of use).

**Figure 3:**
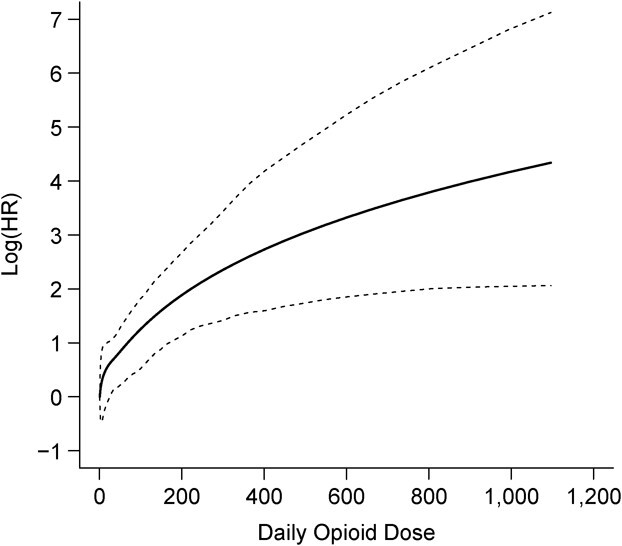
Nonlinear effect of current log-transformed daily opioid dose and the risk of opioid-related emergency department visits, readmissions, or deaths. Data on opioid use and health-care utilization was collected from patients discharged alive from the McGill University Health Centre (MUHC), Canada, between October 2014 and November 2016. The solid line represents the effect estimate of the nonlinear effect of current log-transformed daily opioid dose and the risk of opioid-related emergency department visits, readmissions, or deaths, and the dashed lines represents the corresponding confidence intervals associated with it. HR, hazard ratio.

**Figure 4:**
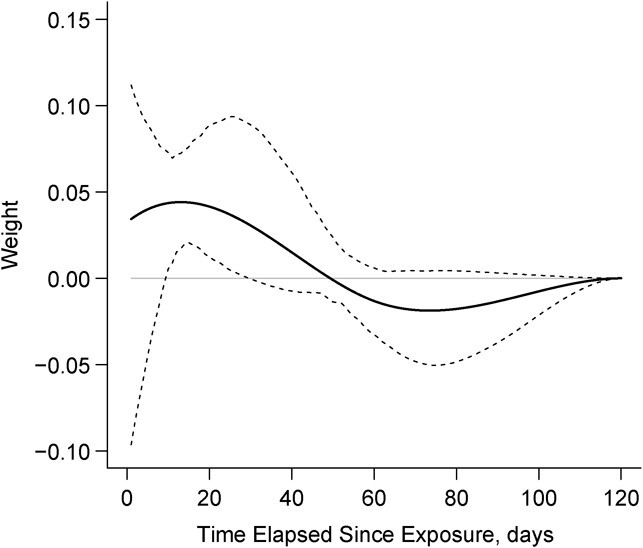
Estimated weight function for current daily opioid use. Data on opioid use and health-care utilization was collected from patients discharged alive from the McGill University Health Centre (MUHC), Canada, between October 2014 and November 2016. The solid line represents the estimated weight function for current daily opioid use, and the dashed lines represents the corresponding confidence intervals associated with it.

**Figure 5:**
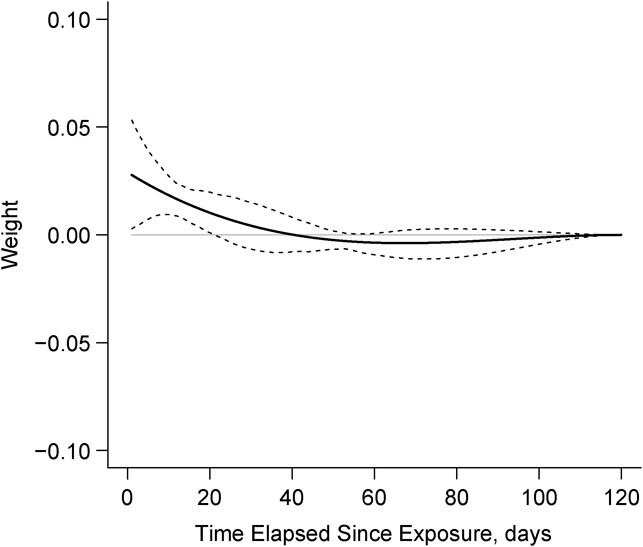
Estimated weight function for current log transformed daily opioid dose. Data on opioid use and health-care utilization was collected from patients discharged alive from the McGill University Health Centre (MUHC), Canada between October 2014 and November 2016. The solid line represents the estimated weight function for current log transformed daily opioid dose, and the dashed lines represents the corresponding confidence intervals associated with it.

The aforementioned nonlinear effects of cumulative duration of use are generally consistent with the results of flexible WCE analyses in Figures 4 and 5. The horizontal axis shows the number of days elapsed (*t*) since the exposure, and the vertical axis shows the corresponding estimated weights, reflecting the relative strength of the association between opioid use “*t* days ago” and the current hazard of opioid-related readmissions/ED visits or death. The estimated weight functions for both past use (Figure 4) and past log-transformed doses (Figure 5) suggest that their impact cumulates over the previous 40 or 50 days, when the weights are positive. Yet the WCE model for the past daily doses fits the data much better (by about 15 AIC points, Table 3), underscoring the importance of accounting for differences in dosage. The corresponding weight function indicates that most recent doses have the highest impact on the current risk of adverse events, whereas doses taken more than a month prior have only very minor effects (Figure 5). The upper part of Table 4 shows how the aHR, relative to no opioid use in the past 120 days, increases with increasing duration of use. Uninterrupted use in the most recent 30 or 60 days is associated with important risks of adverse events or death (aHR = 4.86, 95% CI: 1.56, 6.67, and aHR = 6.68, 95% CI: 2.07, 12.0, respectively). The lower part of Table 4, based on the WCE model for doses, shows that the aHRs associated with doses of 50–120 MME over the past 40 days, relative to no use, are very high (e.g., for 50 MME: aHR = 5.92, 95% CI: 2.46, 14.1). Compared with users of low-dose opioids (25 MME), a daily dose of 90 MME was associated with a 76% increased hazard (aHR = 1.76, 95% CI: 1.33, 2.32) (Table 4).

**Table 4:** Results of Weighted Cumulative Exposure Marginal Structural Cox Models: Hazard Ratio Estimates for the Association Between Specific Patterns of the Past Use and Dosing Regimens of Opioid Exposure and Opioid-Related Health-Care Encounters or Death for Patients Discharged Alive From McGill-Affiliated Hospitals in Montreal Between 2014 and 2016

**Exposure**	**HR**	**95% CI**
Duration of recent opioid use		
No use	1.00	Referent
Previous 10 days	1.47	0.68, 2.35
Previous 20 days	2.72	0.98, 3.87
Previous 30 days	4.86	1.56, 6.67
Previous 60 days	6.68	2.07, 12.0
Opioid dose in the previous 40 days, MME		
0^a^	1.00	Referent
50	5.92	2.46, 14.1
90	7.69	2.81, 20.9
120	8.75	3.00, 25.3
Previous 40-day use, low dose vs. high dose, MME		
25	1.00	Referent
90	1.76	1.33, 2.32

Abbreviations: CI, confidence interval; MME, morphine milligram equivalent; HR, hazard ratio.

## DISCUSSION

Advances in methods to accurately estimate the impact of opioid use are critical for improving guideline recommendations (13). We assessed the associations between opioid dose and duration of use and the risk of potentially opioid-related adverse events by comparing different modeling approaches, including novel flexible multivariable models, to account for the dynamic nature of opioid use, and their cumulative effects. We also considered alternative opioid exposure metrics and compared our results with conventional Cox marginal structural models.

These new analytical techniques offered additional insights regarding possible mechanisms linking past opioid use to adverse events. The weight functions, obtained with the WCE MSM modeling (49, 51), indicated that the most recent opioid use and doses had the highest impact on the current risk, with relatively weak effects of doses taken more than 30 days ago and no impact of opioids taken more than 50 days ago. This finding was corroborated by flexible modeling of nonlinear effects of duration, which indicated risk increases for up to 50–60 days of cumulative past opioid use. However, due to the small number of subjects who accumulated longer duration of use within 1 year after discharge, the corresponding confidence intervals are wide, so that these results should be interpreted with caution. On the other hand, the best-fitting nonlinear model for daily dose showed no evidence of threshold, with the risk increasing continuously with increasing doses, which likely reflects the degree of addiction among “heavy users,” and their resulting high risk of adverse events. To our knowledge, only one previous study used flexible modeling of opioid exposure (59). However, these authors only considered the nonlinear effect of opioid duration and defined the outcome as a self-reported measure of nonmedical use of prescription opioids (59).

Our flexible nonlinear modeling also suggested that, among patients on the same current dose, the hazard decreased with longer continuous opioids use, possibly due to improved tolerance and/or healthy survivor effect (54–56). Selective tolerance is a clinical reality described in the literature, where patients develop tolerance to different opioid-related side effects at a different rate (55, 57, 58). Indeed, the number of side effects a patient experiences is higher for acute compared with chronic opioid administration (57). Previous studies that reported increased risk of adverse events with longer durations focused on more severe outcomes such as opioid-related overdose, abuse, and death (60–62). The risk for these events increases as tolerance develops due to the increased opioid dose required to achieve the same level of analgesia (57, 63).

Identification of the correct exposure model in a context of dynamic and complex treatment regimens could help policy makers rationalize opioid-prescribing practices, which could potentially result in improved benefits/harms ratio. Our findings should be a reassuring addition to the evidence base for clinicians: The data provides support that prescribing and escalating doses above the recommended guidelines should be avoided, and close monitoring of patients who have used opioids for longer periods of time is needed.

Among the strengths of this study, we linked multiple data sources to harness detailed information on multiple potential confounders, to help improve the internal validity of our analyses. In addition, the use of Cox MSMs with inverse probability weighting allowed us to accurately model the associations of interest while considering the dynamic pattern of individual treatment regimens, and accounting for a patient’s medical history, which might have partly affected their treatment changes, and medication-taking behavior. Moreover, we applied a flexible spline-based method, adapted for Cox MSM analyses (51), for modeling: 1) cumulative effects of time-dependent opioid exposures, weighted by the recency of the exposure, as well as 2) nonlinear of opioid dose (45). Finally, unlike other pharmacoepidemiologic studies, we provided information derived from patients’ interviews on the actual consumption of opioids within the immediate 30-day postdischarge period, a critical time window for assessing pain control and its impact on long-term postdischarge outcomes associated with pain treatment (64).

Some limitations of our work should be recognized. First, our study relied on data that are almost 5 years old and may be less relevant to the current clinical and policy environment. Of note, despite the publication of the Centers for Disease Control and Prevention (18) and other clinical practice guidelines for opioid therapy (38, 65) in the past few years, studies suggest that less than 50% of pain management in primary care settings is compliant with these guidelines (66, 67). While the prevalence of high-dose (>200 MME) opioid prescribing has decreased, the rates of opioid-related hospitalizations and ED visits, especially among people aged 55 years or older, have increased, simultaneously with an increased use of high-potency opioids (68, 69). Indeed, in our analyses most of the dispensations were for high-potency hydromorphone and oxycodone. In addition, Quebec has the lowest rate of hospitalizations related to opioid overdose among all jurisdictions across Canada, possibly due to lower opioid doses dispensed (70). Third, by using a broad outcome, we could include events not related to opioid exposure. However, it was not possible to limit our analyses to opioid-specific events, which were very rare in our cohort. Moreover, medication-related adverse events are likely vastly underascertained; in one study, 50% failed to be identified as medication-related (71). Thus, when it comes to recording events such as medication adverse events, diagnostic ascertainment is a well-established problem. Nevertheless, possible outcome misclassification, due to inclusion of events not caused by opioids, would bias the estimated associations toward the null and reduce the statistical power. This combined with a moderate number of adverse events (241) in our cohort implies that our inability to demonstrate some associations may reflect limited statistical power and precision. Yet even if some individual outcomes are misclassified, the increased hazard demonstrated in some of our analyses is likely attributed to higher opioid exposure.

As in all observational studies, there is the risk of unmeasured confounding. However, by including only patients with at least 1 opioid dispensation, we reduce concerns about potential bias due to confounding by indication (72, 73). Last, whereas our analyses failed to demonstrate further risk increases with cumulative duration of opioid use continuing to increase beyond 100 days, the corresponding estimates were very imprecise. This issue requires further studies, possibly using data sources from multiple health-care systems, to replicate our findings in a larger cohort with longer follow-up.

In conclusion, flexible modeling of recency-weighted cumulative opioid use/dose and nonlinear effects of current dose allowed us to illustrate how careful analyses that account for dose, duration, and timing of past exposures may improve the model’s fit to data and enhance our understanding of the mechanism underlying potential adverse events of opioid exposures.

## Supplementary Material

Web_Material_kwad115
