# Supplementary material for: Comparison of Different Modeling Approaches for Prescription Opioid Use and Its Association With Adverse Events
Source: Am J Epidemiol. 2023 May 15;192(9):1592–603. doi: 10.1093/aje/kwad115 (PMC10472496; doi:10.1093/aje/kwad115)
Supplement: Web_Material_kwad115 [file web_material_kwad115.pdf]

Web Material

# Comparison of Different Modeling Approaches for Prescription Opioid Use and Its Association With Adverse Events

Siyana Kurteva, Michal Abrahamowicz, Marie-Eve Beauchamp, and Robyn Tamblyn

Table of Contents


| | |
|---|---|
| Web Figure 1 | 2 |
| Web Table 1 | 3 |
| Web Appendix 1 | 8 |
| Web Table 2 | 9 |
| Web Figure 2 | 11 |
| Web Table 3 | 12 |
| Web Figure 3 | 13 |
| Web Figure 4 | 14 |
| References | 15 |

**Web Figure 1.** Flowchart of eligible patients.

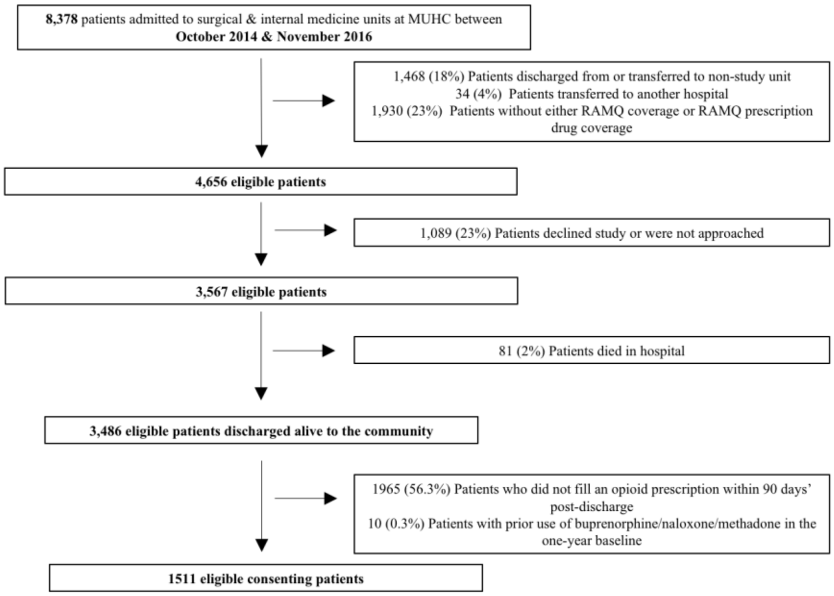

**Web Table 1.** Description of available data on drug, patient, provider and system level characteristics

| | Description | Measurement | Timing of Measurement | Functional Form |
|---|---|---|---|---|
| Opioid-related Characteristics | | | | |
| *Opioid Dispensations* | | | | |
| ATC code | Anatomical Therapeutic Chemical Classification System code used to identify opioids and other concurrent medications that the patient is taking<br><br><u>Opioids ATC Included: N02A, R05DA</u> | RAMQ prescription claims. | In the community one year prior to admission and one year post-discharge | N/A |
| Dose | The daily amount of drug taken by patient was calculated based on information about the number of tablets prescribed, strength and number of days' supply; daily dose was converted to milligram morphine equivalents to facilitate comparison across opioids | From RAMQ prescription claims | In the community one year prior to admission and one year post-discharge | Continuous, categorical, time-varying |
| Duration | The days' supply on the drug claim as entered by the pharmacist | From RAMQ prescription claims | In the community one year prior to admission and one year post-discharge | Continuous, categorical, time-varying |
| Type of opioid | Type of opioid ingredient; e.g.: hydromorphone, oxycodone, morphine, fentanyl, etc. | From RAMQ prescription claims | One year post-discharge | Categorical, time-varying |
| *Opioid Administration in Hospital* | | | | |
| ATC code | Anatomical Therapeutic Chemical Classification System code used to identify administered opioids | Hospital pharmacy | In hospital | Categorical |
| *Opioid Prescription at Hospital Discharge* | | | | |
| Status of opioid medication | Continued or stopped from community, or newly prescribed at discharge | From patient chart | At hospital discharge | Categorical, time-fixed |

| | | | | |
|---|---|---|---|---|
| Reason for opioid prescribing | Pain-related including having had surgery as well as other diagnoses such as having insomnia or anxiety as recorded during the hospitalization | From patient chart | In-hospital | Categorical, time-fixed |
| Presence of a multi-modal pain management regimen | The opioid prescription at hospital discharge as part of multi-modal pain treatment regimen | From patient chart | At hospital discharge | Categorical, time-fixed |
| Patient-reported adherence to opioid prescription given at hospital discharge | Whether patient takes the medication as prescribed or deviates from the prescription posology (e.g.; medication taken less or more often than directed to patient due to pain complaints, complications, side-effects, etc.) | RAMQ prescription claims to determine whether opioid was filled post-discharge; patient interview to assess if patients are taking the drugs as prescribed | 30-days post-hospital discharge | Categorical, time-fixed |
| Opioid-related Characteristics | | | | |
| *Demographics* | | | | |
| Age | | From patient chart | Admission to hospital | Continuous, time-varying |
| Sex | Male, Female | From patient chart | Admission to hospital | Binary, time-fixed |
| Drug insurance status | E.g.: Full copay, partial copay, no copay; serves as proxy for socio-economic status | From RAMQ drug programs | Admission to hospital | Categorical, time-fixed |
| *Co-Existing Illnesses* | | | | |
| History of mental health conditions | E.g.: Anxiety, depression, psychiatric diagnosis, mood disorder, and post-traumatic stress disorder | ICD-9 from RAMQ medical services and ICD-10 codes from hospitalization data | In community one year prior to admission, in hospital, post-discharge | Binary per condition, time-varying |
| Pain syndromes | E.g.: Chronic back pain, back and neck pain, back disorder, arthritis, migraine, headache, fibromyalgia, fracture | ICD-9 from RAMQ medical services and | In community one year prior to admission, in | Binary per condition, time-varying |

| | | | | |
|---|---|---|---|---|
| | | ICD-10 codes from hospitalization data | hospital, post-discharge | |
| Health conditions Associated with abuse | E.g.: Alcohol abuse, drug abuse | From patient chart. Also from RAMQ medical series and prescription claims | In community one year prior to admission, in hospital, post-discharge | Binary per condition, time-varying |
| Tobacco use | Patient-reported history of tobacco use | From hospital charts | At admission | Binary, time-fixed |
| Cancer diagnosis | E.g.: Metastatic, non-metastatic, lymphoma | ICD-9 from RAMQ medical services and ICD-10 codes from hospitalization data | In community one year prior to admission, in hospital, post-discharge | Binary per condition, time-varying |
| Other comorbidities | E.g.: Acute MI, cerebrovascular diseases, chronic kidney, COPD, diabetes, heart failure, hypertension, ischemic heart disease, liver, obesity | ICD-9 from RAMQ medical services and ICD-10 codes from hospitalization data | In community one year prior to admission, in hospital, post-discharge | Binary per condition, time-varying |
| *Drug and Healthcare Utilization* | | | | |
| Use of potential interacting drugs increasing the risk of opioid misuse | E.g.: Selective serotonin reuptake inhibitors, other antidepressants, benzodiazepines, other antipsychotic drugs, central nervous system depressants, psychotropic medication | ATC codes, DIN, Generic Drug name used to extract information from RAMQ prescription claims, hospital data, patient chart. | In community one year prior to admission, in-hospital, post-discharge | Binary per drug, time-varying |
| Use of non-opioid pain medications | E.g.: NSAIDS, COX-2, Acetaminophen, gabapentin, anti-migraine medications, muscle-relaxants, other anti- | ATC codes, DIN, Generic Drug name used to extract information | In community one year prior to admission, in-hospital, post-discharge | Binary per drug, time-varying |

| | | | | |
|---|---|---|---|---|
| | inflammatories and anti-rheumatoid medications | from RAMQ prescription claims, hospital data, patient chart. | | |
| Number of emergency department (ED) visits and hospitalizations | Total number of ED visits and hospitalizations | From RAMQ prescription claims and hospital data | One year prior to hospital admission & one year post-discharge | Categorical, continuous, time-varying |
| *Measures of Care Continuity* | | | | |
| Number of physicians | Number of unique physicians that prescribed an opioid medication to a patient in the year post hospital admission | From RAMQ medical services | One year prior to hospital admission & one year post-discharge | Categorical, continuous, cumulative time-varying |
| Number of dispensing pharmacies | Number of unique pharmacies that a patient has opioid medications dispensed at in the one year post to hospital admission | From RAMQ medical services | One year prior to hospital admission & One year post-discharge | Categorical, continuous, cumulative time-varying |
| *Other Patient Drug Behavior Characteristics* | | | | |
| Time since hospital discharge | The time elapsed between patent's hospital discharge and their first opioid dispensation | From RAMQ prescription claims and hospital data | One year post-discharge | Continuous, time-fixed |
| Discontinuation of opioid use | Recent discontinuation of opioid use in the past two weeks | From RAMQ medical services | One year post-discharge | Categorical, time-varying |
| Daily opioid dose increase | Recent increase in the daily opioid use in the past two weeks | From RAMQ medical services | One year post-discharge | Categorical, time-varying |
| Add-on opioid | Recent add-on of another opioid type in the past two weeks | From RAMQ medical services | One year post-discharge | Categorical, time-varying |
| *In-hospital Characteristics* | | | | |
| Hospital patient is admitted to | Montreal General or Royal Victoria hospital | From hospital chart | Upon admission to the hospital | Binary, time-fixed |
| Hospital unit the patient is admitted to | Medical or surgical unit | From hospital chart | Upon admission to the hospital | Binary, time-fixed |
| Reason for index hospital admission | Reasons were classified as opioid-related if patient presented to the hospital for an opioid-related disorder, poisoning by opioids, or fractures | From hospitalization data | During the hospital stay | Binary, time-fixed |

| | | | | |
|---|---|---|---|---|
| Discharge Destination | Home community, long term care | Patient chart | Upon discharge | Binary, time-fixed |
| RightRx patients | Patients, who were part of the initial randomized controlled trial | Patient chart | Upon discharge | Binary, time-fixed |

**Web Appendix 1.** Codes Used for Drug Classification and Rationale for Opioid Dose and Opioid Duration of Use Calculations.

ATC codes used to identify opioids: N02A (opioids), R05DA (opium alkaloids and derivatives)

Exclusions: Not all drug forms were included in the analyses. Only patches and tablets of these medications were kept. Injectable, liquid and rectal forms were excluded. Methadone and buprenorphine/naloxone combinations were kept to define subclinical patient populations but were excluded from all dosing/duration calculations as these medications are used to treat addiction and we want to focus on the association of duration/dose of opioids used for pain relief.

The daily dose of each opioid was calculated by first dividing the quantity of units dispensed by the prescription duration to determine the number of units per day, and then multiplying the number of units by the strength. To account for concurrent prescriptions, a subsequent dispensation was considered as an early refill if days of overlap were ≤30% of the previous dispensation duration. Otherwise, the opioids were considered to be taken simultaneously. Daily dose of each dispensation was converted to MME doses using the Center for Disease Control Opioid Morphine Equivalent Conversion Factor and the opioid doses determined to be concurrently dispensed were added together.

Cumulative duration of past use assessed the long-term impact of opioids, where the effect on the outcome persisted upon discontinuation: defined as the total number of days exposed, calculated by summing the durations of all dispensations between cohort entry (first opioid dispensation) and a given day during the follow-up. Cumulative users represented patients who used opioids only when needed, thus accumulating use over time. On the other hand, we assessed continuous duration where the effect of opioids accumulated by dispensation supply but the risk returned to baseline after discontinuation. Continuous duration was defined similarly but was allowed to increase only during the periods of uninterrupted use and was reset to zero if there was a gap of >5 days between subsequent dispensations.

**Web Table 2.** Opioid Morphine Equivalent Conversion Factor[a]

| Drug Name | Conversion Factor |
|---|---|
| Buprenorphine patch[b] | 12.6 |
| Buprenorphine tab or film | 10 |
| Butorphanol | 7 |
| Codeine | 0.15 |
| Dihydrocodeine | 0.25 |
| Fentanyl buccal or SL tablets, or lozenge/troche[c] | 0.13 |
| Fentanyl film or oral spray[d] | 0.18 |
| Fentanyl nasal spray[e] | 0.16 |
| Fentanyl patch[f] | 7.2 |
| Hydrocodone | 1 |
| Hydromorphone | 4 |
| Levorphanol tartrate | 11 |
| Meperidine hydrochloride | 0.1 |
| Methadone | 3 |
| Morphine | 1 |
| Nalbuphine | 1 |
| Opium | 1 |
| Oxycodone | 1.5 |
| Oxymorphone | 3 |
| Pentazocine | 0.37 |
| Tapentadol | 0.4 |
| Tramadol | 0.1 |

[a] Centers for Disease Control and Prevention, Atlanta, GA, May 2014 (1).

[b] The MME conversion factor for buprenorphine patches is based on the assumption that one milligram of parenteral buprenorphine is equivalent to 75 milligrams of oral morphine and that one patch delivers the dispensed micrograms per hour over a 24-hour day. Example: 5 ug/hr buprenorphine patch * 24 hrs = 120 ug/day buprenorphine = 0.12 mg/day buprenorphine = 9 mg/day oral morphine milligram equivalent. In other words, the conversion factor not accounting for days of use would be 9/5 or 1.8. However, since the buprenorphine patch remains in place for 7 days, we have multiplied the conversion factor by 7 (1.8 X 7 = 12.6). In this example, MME/day for four 5 μg/hr buprenorphine patches dispensed for use over 28 days would work out as follows: Example: 5 ug/hr buprenorphine patch * (4 patches/28 days) * 12.6 = 9 MME/day. (2)

[c] The MME conversion factor for fentanyl buccal tablets, sublingual tablets, and lozenges/troche is 0.13. This conversion factor should be multiplied by the number of micrograms in a given lozenge/troche.

[d] The MME conversion factor for fentanyl film and oral spray is 0.18. This reflects a 40% greater bioavailability for films compared to lozenges/tablets and 38% greater bioavailability for oral sprays compared to lozenges/tablets.

[e] The MME conversion factor for fentanyl nasal spray is 0.16, which reflects a 20% greater bioavailability for sprays compared to lozenges/tablets.

[f] The MME conversion factor for fentanyl patches is based on the assumption that one milligram of parenteral fentanyl is equivalent to 100 milligrams of oral morphine and that one patch delivers

the dispensed micrograms per hour over a 24 hour day. Example: 25 ug/hr fentanyl patch * 24 hrs= 600 ug/day fentanyl = 60 mg/day oral morphine milligram equivalent. In other words, the conversion factor not accounting for days of use would be 60/25 or 2.4. However, sincethe fentanyl patch remains in place for 3 days, we have multiplied the conversion factor by 3 (2.4 X 3 = 7.2).
In this example, MME/day for ten 25 µg/hr fentanyl patches dispensed for use over 30 days would work out as follows: Example: 25 ug/hr fentanyl patch * (10 patches/30 days)* 7.2 = 60 MME/day.

**Web Figure 2.** Estimated weights function showing the association between current daily opioid use during the entire one-year follow-up period and the current hazard of adverse events.

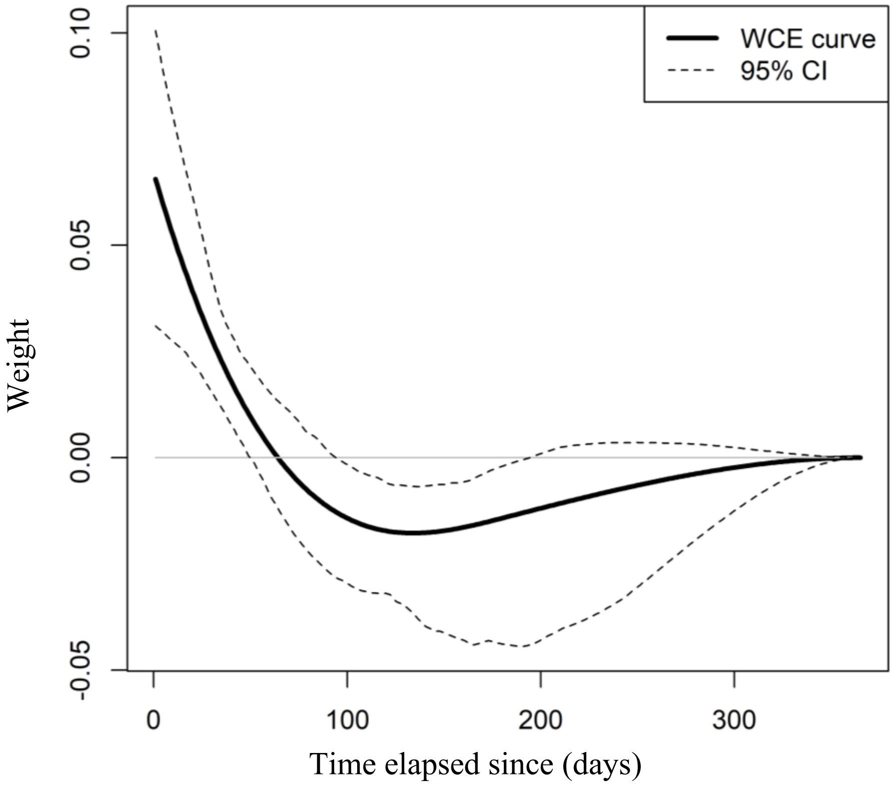

**Web Table 3.** Comparison of goodness of fit in sensitivity analyses for flexible non-linear (NL) MSM additionally adjusting for the non-linear effect of MME current daily dose (log-transformed), with alternative time-varying opioid exposure metrics.

| **Opioid Exposure Metric** | **Statistical Model** | **AIC** |
|---|---|---|
| *Cumulative Duration of Use* | Flexible non-linear (NL) MSM, no adjustment daily dose | 2584.0 |
| | Flexible non-linear (NL) MSM, with adjustment daily dose | 2549.6 |
| *Continuous Duration of Use* | Flexible non-linear (NL) MSM, no adjustment daily dose | 2596.0 |
| | Flexible non-linear (NL) MSM, with adjustment daily dose | 2531.0 |

**Web Figure 3.** Non-linear effect of **cumulative duration of opioid use** and the risk of opioid-related emergency department visits, re-admissions or deaths, additionally adjusted for the non-linear effect current MME daily dose (log-transformed).

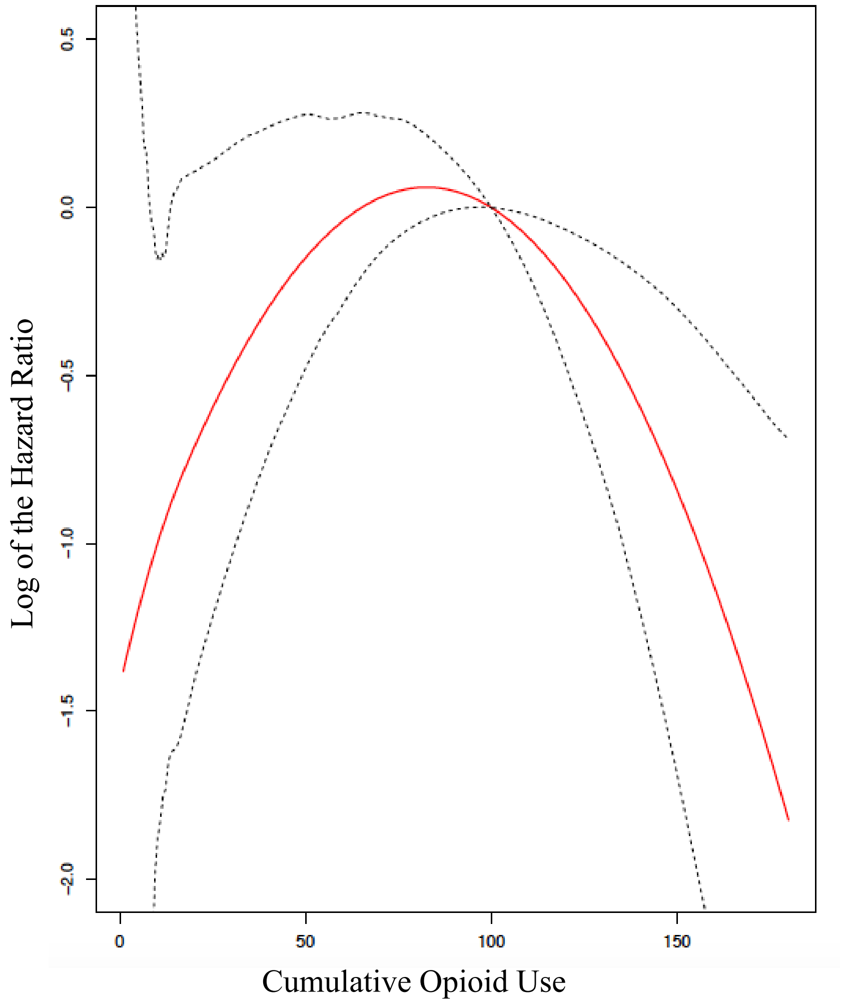

**Web Figure 4.** Non-linear effect of **continuous duration of opioid use** and the risk of opioid-related emergency department visits, re-admissions or deaths, additionally adjusted for the non-linear effect current MME daily dose (log-transformed).

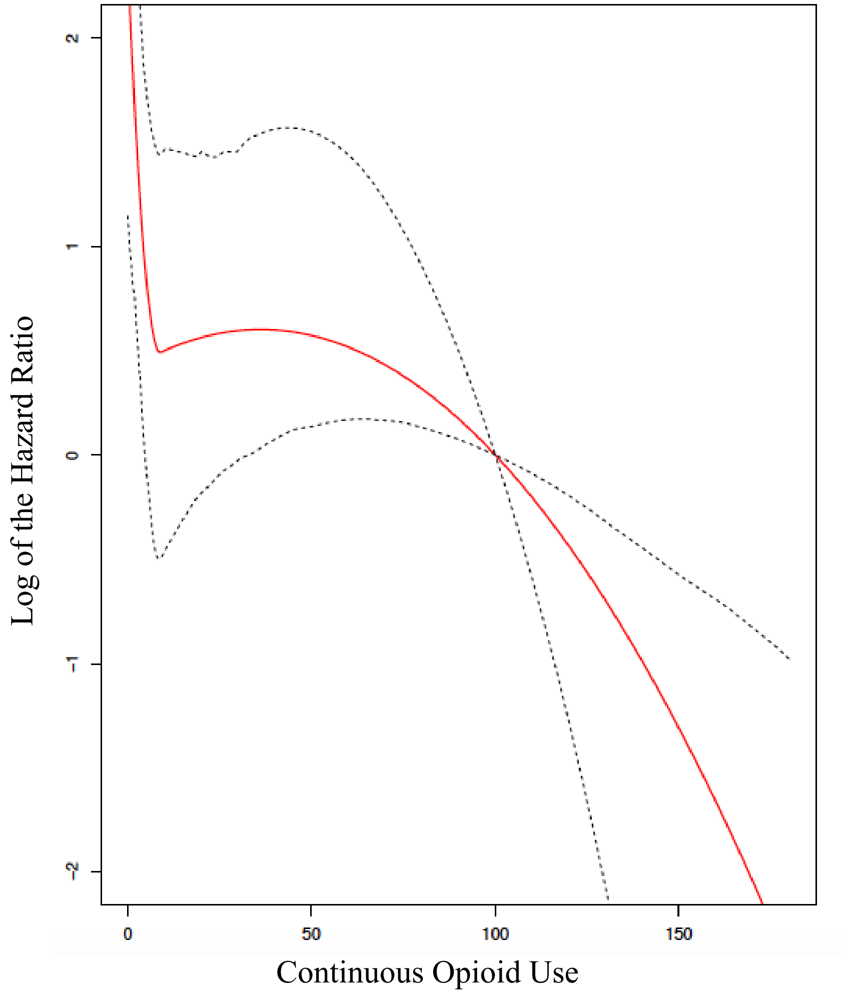

References


1) Centers for Medicare & Medicaid Services. Opioid Oral Morphine Milligram Equivalent (MME) Conversion Factors. https:\\www.cms.govMedicarePrescription-Drug-CoveragePrescriptionDrugCovContraDownloadsOpioid-Morphine-EQConversion-Factors-vFeb-.pdf. Accessed: September 5, 2019
2) Svendsen, K., Borchgrevink, P., Fredheim, O., Hamunen, K., Mellbye, A., & Dale, O. (2011). Choosing the unit of measurement counts: the use of oral morphine equivalents in studies of opioid consumption is a useful addition to defined daily doses. Palliative Medicine, 25(7), 725–732. http://doi.org/10.1177/0269216311398300
